# Clinical Assessment of Altered Eating Behaviors in People with Obesity Using the EBA-O Questionnaire

**DOI:** 10.3390/nu17071209

**Published:** 2025-03-30

**Authors:** Vittorio Oteri, Laura Contrafatto, Gaetano Maria Santoro, Ignazio Barca, Andrea Tumminia, Federica Vinciguerra, Lucia Frittitta, Francesco Frasca, Laura Sciacca, Roberto Baratta

**Affiliations:** 1 Endocrinology Section, Department of Clinical and Experimental Medicine, Garibaldi-Nesima Hospital, University of Catania, 95122 Catania, CT, Italy; santoro.gaetano1997@gmail.com (G.M.S.); ignazio.barca94@gmail.com (I.B.); frascafranco@gmail.com (F.F.); laura.sciacca@unict.it (L.S.); 2 Department of Clinical and Experimental Medicine, University of Catania, 95122 Catania, CT, Italy; l.c.contrafatto@gmail.com (L.C.); vinciguerrafederica@gmail.com (F.V.); lucia.frittitta@unict.it (L.F.); 3 Endocrine Unit, Garibaldi-Nesima Hospital, 95122 Catania, CT, Italy; andreatumminia82@gmail.com (A.T.); rob.baratta@gmail.com (R.B.); 4 Diabetes and Obesity Center, Garibaldi-Nesima Hospital, University of Catania, 95122 Catania, CT, Italy

**Keywords:** eating disorders, overweight, obesity, BMI, night eating, food addiction, sweet eating, hyperphagia, binge eating

## Abstract

**Background/Objectives:** Over the past decade, numerous studies have explored the bidirectional relationship between obesity and mental health, mainly eating disorders (EDs). This study aimed to assess the prevalence and characteristics of altered eating behaviors (AEBs) in a cohort of people with obesity (PwO) using the validated Eating Behaviors Assessment for Obesity (EBA-O). **Methods:** We conducted a cross-sectional study from May 2023 to April 2024, recruiting consecutive PwO seeking weight loss. Participants completed the 18-item EBA-O questionnaire, which focuses on five primary eating behaviors: night eating, food addiction, sweet eating, hyperphagia, and binge eating. Unlike other validated tools, the EBA-O is specifically designed to capture these behaviors in PwO and is easy for patients to self-administer. We also collected sociodemographic and clinical data. **Results:** A total of 127 participants were included (76 women, median age 52 years, median BMI 42.9 kg/m^2^). We found a significant prevalence of AEBs: 33.1% for sweet eating, 23.6% for hyperphagia, 15.7% for food addiction, 14.2% for binge eating, and 7.1% for night eating. The EBA-O scores correlated positively with BMI (r = 0.201, *p* = 0.024) and increased across BMI categories (*p* = 0.001). Males had higher scores for night eating and hyperphagia (*p* = 0.01), and active smokers had higher hyperphagia scores (*p* = 0.043) than ex-smokers and non-smokers. The night eating scores were inversely correlated with sleep hours (r = −0.197, *p* = 0.026), and food addiction was positively correlated with age (r = 0.261, *p* = 0.003); conversely, hyperphagia (r = −0.198, *p* = 0.025) and binge eating (r = −0.229, *p* = 0.010) were inversely correlated with age. PwO without diabetes had higher scores for food addiction (*p* = 0.01) and binge eating (*p* = 0.004) compared to those with diabetes. **Conclusions:** These results highlight the potential to characterize PwO based on their AEBs, offering new opportunities to tailor treatment strategies for PwO by targeting specific eating behaviors.

## 1. Introduction

Obesity is one of the most complex diseases affecting our century and all developed countries. It is a complex, chronic disease with multifactorial causes, including excessive body fat accumulation, leading to various health issues such as type 2 diabetes, cardiovascular disease, hypertension, poor quality of life, and increased mortality [1]. Therapeutic choices should be based on a multidisciplinary approach, including nutritional and psychological assessments, physical activity, and medication management [2].

The most common method to assess obesity is the body mass index (BMI), which the World Health Organization (WHO) defines as obesity when scoring 30 or greater [3]. However, there is a growing movement to redefine obesity as Adiposity-Based Chronic Disease (ABCD) [4]. This new classification could overcome the BMI’s limitations, like its lack of specific criteria for race and age, as well as the medical condition heterogeneity associated with obesity, and would base diagnosis upon three factors: etiology, adiposity grade, and health risks [5]. Another proposed model outlines preclinical and clinical obesity, with the latter being defined as a chronic, systemic illness with altered organ and tissue function due to excess adiposity [6].

The major factors contributing to this condition include hypercaloric food availability and excessive intake, oversized food portions, reduced physical activity, stress, poor sleep, medical conditions (e.g., Cushing’s syndrome, hypothyroidism), genetics, and mental health. Twin studies have shown a moderate correlation between genetic factors and the development of obesity traits [7]. For example, among others, the SNP rs79817709, located in the 3′UTR of the KEAP1 gene, is biologically linked to obesity due to its role in inhibiting the Nrf2 protein, which affects insulin resistance, adipogenesis, and adipocyte differentiation [8].

Research increasingly underscores the complex bidirectional relationship between obesity and mental health disorders. For example, depression was found to be associated with an increased risk of obesity in women and, conversely, women with obesity had a moderately increased risk of depression during the follow-up period than women without obesity [9].

Mood disorders (such as depression and anxiety), bipolar disorder, and schizophrenia are often strictly associated with obesity [10,11]. Factors such as medication side effects, unhealthy coping mechanisms like emotional eating, and socioeconomic disparities contribute to weight gain in this population [12,13].

In this scenario, obesity should be viewed not only physically but also psychologically. Eating disorders (EDs) (DSM-5) [14] are linked to obesity. Studies show a significant prevalence of EDs (e.g., Binge Eating Disorder and Night Eating Syndrome) and pathological eating behaviors (e.g., hyperphagia, binge eating, food addiction, and sweet eating) among people with obesity (PwO) [15]. As of 2022, the WHO estimates that 43% of adults worldwide are overweight, and 16% have obesity [16]. Among PwO, 10–15% have BED, increasing to 30% of those with severe obesity and 60–70% among those seeking bariatric surgery [17]. Moreover, NES in PwO was reported with a frequency between 6 and 16% [18].

Vice versa, a history of overweight or obesity may be a risk factor for the development of an ED through multifactorial triggers such as societal weight stigma [19], family dynamics, and the overconsumption of obesogenic foods [20]. Conversely, EDs play an important role in the perpetuation and chronicization of obesity, worsened by body dissatisfaction. In addition, this causal bidirectional relation has also been confirmed using Mendelian randomization analysis [21].

Interestingly, for all the co-diagnoses of obesity and mental health conditions, except for psychosis-spectrum conditions, obesity was often the first-occurring diagnosis [22]. The key measures to tackle this double health burden surely include screening interventions for obesity and psychiatric conditions, nutritional advice for the general population, the implementation of social support services, and access to pharmacological and non-pharmacological treatments [23].

Despite this evidence, several critical research gaps remain. Indeed, there is a limited availability of concise, validated instruments specifically tailored to the detection of altered eating behaviors (AEBs) in PwO. For this reason, we witness a persistent underdiagnosis and inadequate clinical management of specific EDs among PwO. Moreover, we need a better understanding of how sociodemographic (e.g., sex, age, smoking status, and sleep duration) and clinical factors (e.g., diabetes and chronic kidney disease) influence the occurrence and severity of AEBs.

Recently, the “Eating Behaviors Assessment for Obesity (EBA-O)” [24] was developed to assess AEBs (i.e., night eating, food addiction, sweet eating, hyperphagia, and binge eating). These AEBs are defined as repeated or constant disruptions of eating behaviors that result in a modified consumption of food and can harm physical and psychological health [24]. Unlike other validated questionnaires about AEBs, EBA-O is short, easy to use, and can be self-administered by patients, making it an effective tool for identifying AEBs in PwO.

We aim to use the EBA-O to assess the prevalence of AEBs and their association with sociodemographic and clinical characteristics in a cohort of PwO admitted to our endocrine unit in order to address the aforementioned existing research gaps.

## 2. Materials and Methods

### 2.1. Study Design and Setting

From May 2023 to April 2024, we enrolled consecutive PwO admitted as inpatients for the diagnosis and treatment of obesity and/or diabetes complications at the Endocrinology Unit of the Garibaldi-Nesima Hospital in Catania (Italy).

We adhered to the Strengthening the Reporting of Observational Studies in Epidemiology (STROBE) guidelines when reporting our cross-sectional study [25].

This study was conducted in accordance with the 1964 Declaration of Helsinki and its subsequent amendments. The local ethics committee (Catania 2) approved the study protocol (protocol N° 94/CECT2). All participants provided written informed consent before any procedure took place.

### 2.2. Inclusion and Exclusion Criteria

The inclusion criteria were the following: age above 18 years old; BMI ≥ 30 kg/m^2^. The exclusion criteria encompassed the following: a cognitive impairment that prevented a person from completing the assessment; the confirmed diagnosis of a current eating disorder in the last three months; or the use of therapies for the treatment of mental health disorders.

### 2.3. Outcomes

The primary outcome was to study the prevalence of AEBs through the EBA-O questionnaire. The secondary outcome was to assess the relationships between AEBs and the sociodemographic, anthropometric, and clinical characteristics of the participants.

### 2.4. Data Collection

Data on the patients’ sociodemographic status, anthropometric measurements, and clinical characteristics were collected. Patients answered the EBA-O after an endocrinological visit [24]; as above described, it is an 18-item comprehensive validated questionnaire designed to evaluate the 5 major AEBs that contribute to obesity. Each item is scored on a Likert scale from 0 (never) to 7 (always), with higher scores indicating more severe disordered eating behaviors. A total EBA-O score ≥ 4 suggests the presence of clinically relevant pathological eating behavior; factor scores ≥ 4 (e.g., night eating, sweet eating, …) indicate the specific dysfunctional eating behavior. The questionnaire was self-administered, as we allowed the patient to complete the questionnaire independently.

### 2.5. Statistical Analysis

Statistical analysis was run with SPSS software version 29.0 (IBM Corp. Released 2023. IBM SPSS Statistics for Macintosh, Version 29.0.2.0 Armonk, NY, USA: IBM Corp.). All the variables were tested for normal distribution by visual representation and via the Shapiro–Wilk test. The data are expressed as mean ± standard deviation (SD) for parametric variables, median and interquartile range (IQR) for nonparametric variables, and frequencies and percentages for categorical variables.

To test the relationships between two or more variables we applied Pearson’s Product–Moment Correlation for parametric variables and Spearman’s Rank–Order Correlation for nonparametric variables. An independent samples *t*-test and Mann–Whitney U test were conducted, respectively, to compare the differences in the parametric and nonparametric variables between two independent groups. One-way ANOVA and the Kruskal–Wallis H test were used, respectively, to compare differences in the parametric and nonparametric variables across three or more independent groups.

A multivariable analysis was conducted to correct for patients’ main sociodemographic, anthropometric, and clinical characteristics. To predict the value and the categorization of a variable, we used, respectively, multiple and binomial logistic regression, reporting the B coefficient, or odds ratio (OR), and the 95% confidence interval (CI). In multiple regression, the dependent variable was the EBA-O score, whereas in logistic regression, it was the categorization of an EBA-O score as ≥4. For both analyses, the predictor variables included in the model were sex, age, BMI, daily sleep duration, smoking status, presence of hypertension, diabetes, and chronic kidney disease (CKD).

For all analyses, an alpha value of 5% was used.

A power analysis was conducted, using G*Power software (version 3.1) based on the methodologies described by Faul et al. [26], to assess the statistical power of our primary endpoint: the total EBA-O questionnaire score. With a medium effect size (Cohen’s d = 0.5), an alpha value of 5%, and equal-sized subgroups, our total sample size (n = 127) achieved approximately 80% power, sufficient to reliably detect moderate differences in altered eating behaviors across demographic and clinical subgroups.

## 3. Results

### 3.1. Descriptive Statistics

**Figure 1:**
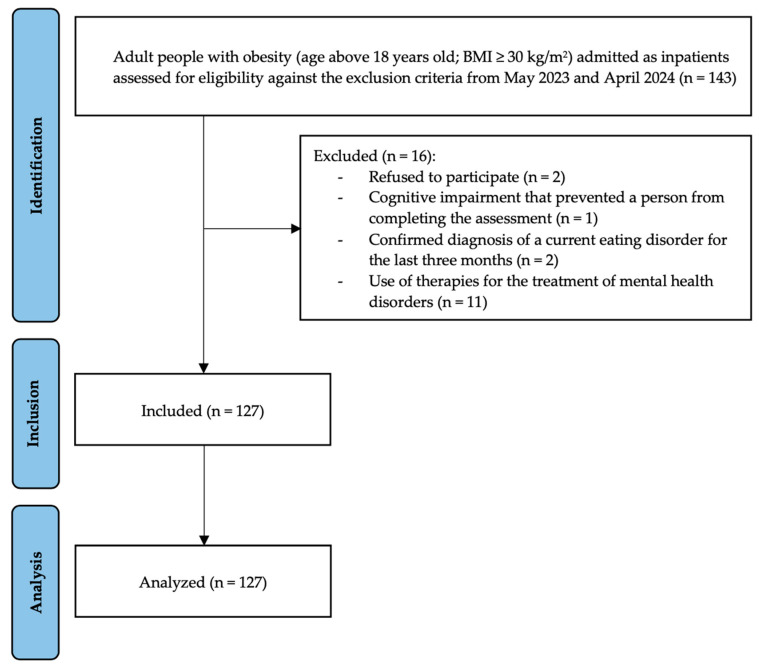
Flow diagram of participants.

The final sample included 127 participants, (51 M; 76 F) with a median age of 52 years (IQR = 42.5–62.5). The median BMI was 42.9 kg/m^2^, with most participants having a BMI between 36.4 and 48.1 kg/m^2^. The participants reported an average of 6 h of sleep per night. Most participants (n = 100; 78%) reported no physical activity during the week; a small proportion (n = 9; 7.1%) engaged in 1–2 h of physical activity per week; and the remaining (n = 18; 14.2%) reported more than 2 h of physical activity per week. Overall, 55% of participants were non-smokers, 21% were current smokers, and 24% were former smokers. Figure 1 illustrates the flow diagram of the participants according to the STROBE guidelines. The whole baseline clinical and demographic characteristics of our participants are reported in Table 1, and Table 2 summarizes their main comorbidities.

### 3.2. Prevalence of AEBs According to EBA-O

The prevalence of AEBs, based on a score ≥ 4 in at least one factor of the EBA-O, ranged from 7.1% for night eating to 33.1% for sweet eating. The results of the EBA-O for the overall sample are presented in Table 3.

### 3.3. Relationships Between EBA-O Scores and Sociodemographic Factors

Night eating was inversely correlated with hours of sleep (r = −0.197, *p* = 0.026). The food addiction score exhibited a positive correlation with age (r = 0.261, *p* = 0.003) while hyperphagia demonstrated an inverse correlation with both age (r = −0.198, *p* = 0.025) and hours of sleep (r = −0.179 *p* = 0.043). Similarly, binge eating was negatively correlated with age (r = −0.229, *p* = 0.010).

The hyperphagia and night eating scores were significantly higher in male compared to female participants (2.67 [0.33–5.33] vs. 1.00 [0.00–2.67], *p* = 0.010, for hyperphagia; 1.50 [0.00–2.50] vs. 0.00 [0.00–1.69], *p* = 0.031, for night eating); nonetheless, females exhibited a higher prevalence of night eating (9.2% F vs. 3.9% M with a night eating score ≥ 4), whereas hyperphagia was more prevalent in males (43.1% vs. 10.5% of females with a hyperphagia score ≥ 4)

For smoking status, a significant difference was found in the hyperphagia scores, with active smokers demonstrating the highest propensity for overeating compared to ex-smokers and non-smokers, respectively (2.33 [1.17–4.75] vs. 1.00 [0.00–3.67] vs. 1.00 [0.00–3.08], *p* = 0.043).

In the multivariable analysis (Table 4 and Table 5), the total EBA-O score and the night eating score diminished, respectively, by 0.14 units and 0.21 units for every additional hour of sleep (B = −0.14, *p* = 0.038, for total score; B = −0.21, *p* = 0.007, for night eating score). Male individuals had a mean increase of 1.25 units in their hyperphagia score when compared to women (B = 1.25, *p* = 0.001). Additionally, the hyperphagia score decreased by 0.04 units for each additional year of age and by 0.25 units for every extra hour of sleep (B = −0.04, *p* = 0.017; B = −0.25, *p* = 0.009). For every additional hour of sleep, the odds of having a night eating score ≥ 4 diminished by 43% (OR 0.57, 95% CI [0.38–0.86], *p* = 0.007). Similarly, the odds of having a food addiction score ≥ 4 diminished by 24% for every additional hour of sleep (OR 0.76, 95% CI [0.58–0.99], *p* = 0.047). The odds of having a hyperphagia score ≥ 4 diminished by 25% for every additional hour of sleep (OR 0.75, 95% CI [0.56–0.96], *p* = 0.032), and were 9.2 times higher in males compared to females (OR 9.20, 95% CI [3.00–28.22], *p* < 0.001).

### 3.4. Relationships Between EBA-O Scores and Anthropometric Data

The total EBA-O score showed a direct correlation with BMI (r = 0.201, *p* = 0.024). Night eating showed a positive correlation with weight (r = 0.175, *p* = 0.049), whereas food addiction (r = 0.346, *p* < 001) and binge eating (r = 0.239, *p* = 0.007) displayed a positive correlation with BMI. Hyperphagia exhibited an inverse correlation with the weight loss per day during inpatient stay (r = −0.224, *p* = 0.015).

Individuals with higher BMI classifications (Class III compared to Class II and Class I) were more likely to report elevated scores in the total EBA-O score (1.98 [0.95–3.11] vs. 1.57 [0.53–2.40] vs. 1.09 [0.56–1.64], *p* = 0.013), food addiction (1.60 [0.40–3.20] vs. 0.80 [0.00–1.70] vs. 0.00 [0.00–0.80], *p* < 001), and binge eating (1.67 [0.00–3.17] vs. 0.00 [0.00–1.67] vs. 0.00 [0.00–0.50], *p* = 0.001).

In the multivariable analysis (Table 4 and Table 5), the food addiction score increased by 0.05 units for each unit rise in BMI (B = 0.05, *p* = 0.006), while the binge eating score augmented by 0.04 units for each unit increase in BMI (B = 0.04, *p* = 0.030). The odds of having a food addiction score ≥ 4 increased by 6% for every one-unit increase in BMI (OR 1.06, 95% CI [1.01–1.12], *p* = 0.033).

### 3.5. Association Between EBA-O Scores and Clinical Characteristics

Individuals without diabetes showed higher scores for food addiction (1.60 [0.40–3.15] vs. 0.80 [0.00–1.80], *p* = 0.010) and binge eating (1.67 [0.00–3.25] vs. 0.00 [0.00–2.00], *p* = 0.004) than people with diabetes (PwD).

Individuals affected by CKD, when compared to non-CKD participants, showed significantly lower scores in total EBA-O (0.60 [0.46–1.30] vs. 1.68 [0.85–2.92], *p* = 0.014), food addiction (0.0 [0.00–1.20] vs. 1.20 [0.00–2.85], *p* = 0.048), sweet eating (0.67 [0.17–2.50] vs. 3.00 [1.33–4.67], *p* = 0.041), and binge eating (0.00 [0.00–1.17] vs. 0.67 [0.00–2.67], *p* = 0.030).

In the multivariable analysis (Table 4 and Table 5), PwD had a mean decrease of 0.77 units in their binge eating score when compared to non-PwD (B = −0.77, *p* = 0.049). The odds of having a sweet eating score ≥ 4 were diminished by 63% (OR 0.37, 95% CI [0.15–0.92], *p* = 0.033) among PwD.

## 4. Discussion

The results of this study emphasize the importance of addressing both the physical and psychological aspects of obesity, suggesting that, beyond caloric intake and physical activity, emotional and behavioral factors play a crucial role in the obesity pandemic.

The EBA-O questionnaire revealed a significant prevalence of AEBs, including sweet eating, hyperphagia, food addiction, and binge eating, among PwO admitted to our endocrine unit. At least one in fourteen PwO that access our facility have a high probability of having one or more AEBs that are not diagnosed and treated; this number can build up to one in three patients for sweet eating, and one in four for hyperphagia. These findings align with previously published studies that show a strong link between obesity and maladaptive eating behaviors [27,28,29,30,31,32,33]. These data reinforce the need for the systematic use of screening tools, such as the EBA-O questionnaire, in daily clinical practice, as well as subsequent structured behavioral modifications, nutritional education, and psychological support for PwO.

Multivariable analysis revealed that a younger age was a significant predictor of a higher EBA-O hyperphagia score. Moreover, the inverse relationship with age suggests that binge eating and hyperphagia tendencies are more pronounced in younger individuals, which could reflect developmental and lifestyle factors. Younger populations may be more susceptible to environmental triggers, social pressures, or emotional stressors that contribute to binge eating and hyperphagic behaviors. Even though the age range of our sample was not so wide, these findings could also be explained by the evidence that models of obesity that develop in younger people, which may be strongly associated with monogenic etiologies, witness hyperphagia as a distinctive trait [34,35]; polygenic forms of obesity are more influenced by the environment and have a greater prevalence of emotional influences. This finding of distinctive hyperphagia or binge eating behavior in young individuals with obesity, in addition to childhood onset and a familial history of obesity, should alert the clinician to counsel a psychological evaluation and, eventually, perform genetic testing to exclude these important causes of obesity [36,37].

The analysis of sex revealed that females exhibited a higher prevalence of AEBs except for hyperphagia; this was confirmed by the strong finding during the multivariable analysis showing that males’ odds of having an altered EBA-O hyperphagia score were 9.2 times higher compared to females. It is established that males and females have distinct eating patterns, which can contribute to different AEBs [38,39]. For this reason, nutritional counseling and management of the psychopathology of AEBs should be tailored also according to gender.

Among modifiable risk factors, hours of sleep are inversely correlated with the odds of having an AEB such as night eating, hyperphagia, or food addiction. Indeed, as confirmed by multivariable analysis, every additional hour of night sleep lowered the odds of having an altered EBA-O score by 43% for night eating, by 24% for food addiction, and by 25% for hyperphagia. Night eating, for example, is often linked to disrupted circadian rhythms and poor sleep quality: patients with NES are likely experiencing reduced sleep, which may exacerbate their metabolic dysregulation and contribute to weight retention or gain, highlighting the impact of this behavior on sleep patterns [40]. Early psychological and sleep management interventions could improve outcomes for these patients. As demonstrated in children, sleep education and targeted sleep interventions may enhance the effectiveness of obesity treatments. In particular, building a consistent sleep schedule and bedtime routine, eliminating other activities before going to sleep, and monitoring daytime naps and rest times may be used in PwO [41].

It was also observed that individuals with an active smoking habit were more prone to hyperphagia compared to ex-smokers and non-smokers. This finding may suggest a potential link between hyperphagia and anxiety levels, which are often associated with tobacco consumption [42]. Anxiety management evaluation and counseling about smoking cessation or reduction should always be complementary in PwO that exhibit these behavioral patterns. A combination of behavioral interventions (individual or group counseling and behavioral interventions, telephone counseling, and mobile phone-based interventions) and an established pharmacotherapy (nicotine replacement therapy, bupropion hydrochloride sustained release, and varenicline) for smoking cessation is recommended for these individuals [43].

As suspected, the severity of eating behaviors strictly correlated to the severity of obesity. Specifically, we found that one unit increase in BMI conferred a 6% increase in the odds of having an altered EBA-O food addiction score in the multivariable analysis. This result confirmed the well-known association between AEBs and obesity, serving as a warning for the impending comorbidities commonly associated with these conditions [44], as well as for the attrition that could impair weight loss [45]. Obesity and AEBs exacerbate each other in a bidirectional manner, searching for a structured treatment plan that could manage both conditions. In particular, it was demonstrated that PwO who exhibit a greater number of AEBs could also display a more severe psychopathology [46]. The EBA-O questionnaire is a promising screening tool that aids in the recognition of the presence of AEBs and that could speed up a referral to focused care to break the vicious cycle of obesity and the psychopathology of eating.

Bearing a diagnosis of a complex chronic condition, such as diabetes or chronic kidney disease, appears to offer some protection against AEBs. Particularly, our multivariable analysis demonstrated that PwD had a 63% reduction in the odds of having an altered EBA-O sweet eating score. In this scenario, the heightened awareness of living with a chronic disease and its potentially severe complications may serve as a deterrent from engaging in disordered eating behaviors. The patients’ education, provided by physicians and dieticians, plays a crucial role in the influence of chronic disease management of dietary behaviors, stimulating more individuals to adhere to structured eating regimens as part of their treatment. Moreover, the use of pharmacological therapies, and especially of GLP-1 receptor agonists, for the treatment of diabetes and obesity can influence eating behaviors [47]; this aspect needs further clarification to also assess clinical efficacy on the psychopathology pathway of the various eating behaviors.

The EBA-O questionnaire adds complexity to a new or an existing diagnosis of obesity, aiming for a tailored therapeutic approach to ABCD. It is a powerful tool that can provide healthcare professionals with insight into the underlying behavioral patterns, emotional triggers, and cognitive processes related to overeating or unhealthy dietary habits. The purpose and usage of this tool is to screen for specific AEBs that contribute to obesity; indeed, the results may help providers refer their patients to more specialized settings and develop tailored and integrated treatment plans, including dietary changes, medications, and psychological support. Moreover, the EBA-O can be administered over time to track changes in eating behaviors, helping professionals monitor the effectiveness of interventions and adjust accordingly the treatment plan. Lastly, the EBA-O can engage patients in understanding their eating patterns, motivating them to make healthier choices. The administration and interpretation of the single available and validated tests for eating disorders in PwO require a lot of time and specialized figures by teams of experts in this field. The EBA-O questionnaire is reliable and easy to use, is shorter, and can diagnose five different AEBs, allowing clinical interpretation for clinicians without experience in eating disorders as well; furthermore, it is easy to administer, as patients can complete it autonomously [24].

Eating disorders are a complex group of diseases that pose a global health concern and demand appropriate treatment, both addressing the mental (e.g., anxiety, depression, and self-harm behaviors) and physical (e.g., obesity or malnutrition, osteoporosis, and hypothalamic–pituitary axis dysfunctions) health of affected people. Notwithstanding, eating disorders remain an underdiagnosed condition [48]. From 1997 to 2017, the global prevalence of eating disorders experienced an estimated annual percentage change of 0.65. The highest burden was registered in high-sociodemographic-index countries and was higher in females than in males, but the increase was greater in males than in females over the time considered [49].

In this context, mental health plays a crucial role in obesity generation and perpetration. Undiagnosed mental health disorders and AEBs could delay or even totally prevent the attainment of an effective treatment. People with AEBs may exhibit disruptive eating patterns that may counteract and override the long-term health benefits of adhering strictly to a structured, evidence-based nutritional regimen, such as the Mediterranean diet [50,51,52]. This issue becomes critical for PwO and also bears the weight of numerous metabolic and structural complications (such as in our cohort). Future studies should investigate if the AEBs evaluated with the EBA-O questionnaire are linked to distinct metabolic parameters that could affect the development and worsening of obesity-associated complications.

### Limitations

The participants were mainly affected by class III obesity (63.8%, with a median BMI of 42.9 kg/m^2^), as our hospital is a tertiary referral center for obesity, and the setting is dedicated mostly to people with severe and complex obesity who are admitted as inpatients for the diagnosis and treatment of obesity and/or diabetes complications. This limits the generalizability of our findings, and further studies with diverse populations could help clarify these patterns and their applicability across broader contexts.

Interestingly, we did not find any significant relationship between waist circumference and the EBA-O scores. Waist circumference is a key anthropometric measure, complementary to BMI in the evaluation of ABCD, that gives the clinician important information about visceral adiposity and reflects consequent metabolic dysregulations in PwO [53]. Unfortunately, our sample consisted mainly (over 60%) of participants affected by class III obesity, a particular category in which waist circumference measurement may be biased by the patients’ habitus, and its values could not be used reliably for anthropometric purposes, even if continuing to be reliable for cardiovascular risk assessment and to predict obesity-related mortality [54].

Given the intrinsic limitations of our cross-sectional study design, further prospective studies should be conducted to establish our findings, capture important changes that can occur over time, and allow absolute conclusions on the plausible cause–effect relationships between the studied variables. In addition, more focused studies should investigate the usefulness of EBA-O scores to predict key outcomes for PwO, such as weight loss, the effectiveness of pharmacological and non-pharmacological therapies, and the development of obesity-associated complications.

## 5. Conclusions

The findings of our study underscore the impact of AEBs on PwO, assessed with the EBA-O questionnaire. Our findings highlight the need for individualized approaches to obesity treatment, addressing specific eating behaviors; the phenotypization of PwO could allow us to tailor the most appropriate clinical intervention. While caloric intake and physical activity are central to weight management, the psychological and behavioral aspects of eating disorders must also be targeted to ensure long-term success; cognitive-behavioral therapy, interventions to improve sleep and cease smoking, and nutritional education focusing on managing sweet eating and hyperphagia are critical components of a comprehensive obesity treatment strategy.

## Figures and Tables

**Table 1:** Clinical and demographic characteristics of participants.

	n	%
Participants	127	100
Sex		
Female	76	59.8
Male	51	40.2
Age ^§^ (years)	52	42.5–62.5
BMI ^§^ (kg/m^2^)	42.9	36.4–48.1
Obesity class		
I	25	19.7
II	21	16.5
III	81	63.8
Waist circumference ^§^ (cm)	129	118–142
Daily sleep duration ^§^ (hours)	6	4–7
Physical activity per week		
No activity	100	78.7
1–2 h	9	7.1
>2 h	18	14.2
Education level		
Elementary/Middle school		53.5
High School/University		46.5
Occupation		
Housewives		14.2
Unemployed		21.3
Employed		38.6
Retired		21.3
Students		4.7
Smoking habits		
Non-smokers		55.1
Smokers		20.5
Former Smokers		24.4

^§^ Data are presented as median and interquartile range; BMI, body mass index.

**Table 2:** Participants’ main comorbidities.

Comorbidities	Fr	%
Previous bariatric surgery	11	8.7
Musculoskeletal disorders	50	39.4
Respiratory abnormalities at spirometry	23	18.1
OSAS	63	49.6
Liver steatosis	88	69.3
Diabetes	71	55.9
IFG	11	8.7
IGT	22	17.3
Hypertension	85	66.9
Dyslipidemia	81	63.8
Chronic kidney disease	9	7.1
Cancer	9	7.1

OSAS, obstructive sleep apnea syndrome; IFG, impaired fasting glucose; IGT, impaired glucose tolerance.

**Table 3:** EBA-O questionnaire scores and prevalence of AEBs.

Item	Variable	Value
Total EBA-O score	Total EBA-O score, median (IQR)	1.6 (0.8–2.8)
	Number of individuals with score ≥ 4, % (n)	8.7% (11)
	Prevalence of AEB according to gender, %	11.8% F vs. 3.9% M
Night Eating	Night eating score, median (IQR)	0.5 (0.0–2.0)
	Number of individuals with score ≥ 4, % (n)	7.1% (9)
	Prevalence of AEB according to gender, %	9.2% F vs. 3.9% M
Food Addiction	Food addiction score, median (IQR)	(0.0–2.6)
	Number of individuals with score ≥ 4, % (n)	15.7% (20)
	Prevalence of AEB according to gender, %	21.1% F vs. 7.8% M
Sweet Eating	Sweet eating score, median (IQR)	2.7 (1.3–4.7)
	Number of individuals with score ≥ 4, % (n)	33.1% (42)
	Prevalence of AEB according to gender, %	35.5% F vs. 29.4% M
Hyperphagia	Hyperphagia score, median (IQR)	1.3 (0.0–3.5)
	Number of individuals with score ≥ 4, % (n)	23.6% (30)
	Prevalence of AEB according to gender, %	10.5% F vs. 43.1% M
Binge Eating	Binge eating score, median (IQR)	0.3 (0.0–2.5)
	Number of individuals with score ≥ 4, % (n)	14.2% (18)
	Prevalence of AEB according to gender, %	14.5% F vs. 13.7% M

**Table 4:** Multivariable analysis to predict the value of the EBA-O scores.

Variable	Total EBA-O Score		Night Eating		Food Addiction		Sweet Eating		Hyperphagia		Binge Eating	
	B	*p*	B	*p*	B	*p*	B	*p*	B	*p*	B	*p*
Sex	0.23	0.370	0.16	0.606	−0.17	0.615	−0.17	0.692	1.25	**0.001**	0.08	0.836
Age (years)	−0.02	0.066	−0.2	0.110	−0.02	0.260	0.01	0.967	−0.04	**0.017**	−0.03	0.099
BMI (kg/m^2^)	0.02	0.101	0.01	0.513	0.05	**0.006**	0.01	0.990	0.01	0.622	0.04	**0.030**
Daily sleep duration (hours)	−0.14	**0.038**	−0.21	**0.007**	−0.14	0.090	0.08	0.425	−0.25	**0.009**	−0.15	0.100
Smoking status	−0.30	0.053	−0.37	0.051	−0.30	0.135	−0.19	0.452	−0.41	0.073	−0.24	0.276
Hypertension	0.26	0.431	0.29	0.455	−0.17	0.691	0.45	0.395	0.13	0.782	0.57	0.214
Diabetes	−0.26	0.333	0.18	0.590	−0.25	0.471	−0.79	0.074	0.31	0.435	−0.77	**0.049**
CKD	−0.71	0.149	−0.40	0.496	−0.49	0.443	−1.44	0.073	−0.36	0.619	−0.87	0.217

The variables used as predictors in the model are the following: sex, age, body mass index (BMI), daily sleep duration, smoking status, presence of hypertension, diabetes, and chronic kidney disease (CKD). Reference categories for categorical variables: sex, 0 = female, 1 = male; smoking status, 0 = smoker or ex-smoker, 1 = non-smoker; hypertension, 0 = absent, 1 = present; diabetes, 0 = absent, 1 = present; CKD, 0 = absent, 1 = present. Statistically significant results are highlighted in bold.

**Table 5:** Multivariable analysis to predict the categorization of an EBA-O score ≥ 4.

Variable	Total EBA-O Score			Night Eating			Food Addiction			Sweet Eating			Hyperphagia			Binge Eating		
	OR	95% CI	*p*	OR	95% CI	*p*	OR	95% CI	*p*	OR	95% CI	*p*	OR	95% CI	*p*	OR	95% CI	*p*
Sex	0.22	[0.04–1.21]	0.082	0.39	[0.06–2.54]	0.327	0.35	[0.10–1.25]	0.106	1.23	[0.51–2.97]	0.649	9.20	[3.00–28.22]	**<0.001**	1.29	[0.40–4.15]	0.667
Age (years)	0.98	[0.92–1.04]	0.413	0.99	[0.92–1.06]	0.698	0.98	[0.94–1.03]	0.468	0.99	[0.96–1.03]	0.547	0.97	[0.93–1.01]	0.127	0.99	[0.94–1.03]	0.613
BMI (kg/m^2^)	1.00	[0.93–1.08]	0.955	1.05	[0.97–1.14]	0.227	1.06	[1.01–1.12]	**0.033**	1.01	[0.97–1.06]	0.566	1.02	[0.97–1.07]	0.530	1.04	[0.99–1.1]	0.127
Daily sleep duration (hours)	0.81	[0.57–1.15]	0.233	0.57	[0.38–0.86]	**0.007**	0.76	[0.58–0.99]	**0.047**	1.14	[0.91–1.43]	0.249	0.75	[0.56–0.96]	**0.032**	0.97	[0.73–1.29]	0.820
Smoking status	0.40	[0.08–1.97]	0.260	0.21	[0.03–1.62]	0.135	0.68	[0.17–2.64]	0.576	0.66	[0.24–1.85]	0.432	0.40	[0.11–1.39]	0.147	0.55	[0.15–2.08]	0.378
Hypertension	1.95	[0.31–12.3]	0.476	0.93	[0.11–7.69]	0.949	0.60	[0.15–2.37]	0.461	2.01	[0.68–5.92]	0.207	0.69	[0.19–2.46]	0.563	1.24	[0.31–4.93]	0.757
Diabetes	1.98	[0.44–8.82]	0.372	0.89	[0.20–6.29]	0.889	1.29	[0.39–4.29]	0.677	0.37	[0.15–0.92]	**0.033**	1.38	[0.43–4.40]	0.584	0.67	[0.20–2.24]	0.515
CKD	0.00	[0.00–0.00]	0.999	0.00	[0.00–0.00]	0.999	0.00	[0.00–0.00]	0.999	0.29	[0.03–2.77]	0.284	0.97	[0.12–7.62]	0.974	0.00	[0.00–0.00]	0.999

The variables used as predictors in the model are the following: sex, age, body mass index (BMI), daily sleep duration, smoking status, presence of hypertension, diabetes, and chronic kidney disease (CKD). Reference categories for categorical variables: sex, 0 = female, 1 = male; smoking status, 0 = smoker or ex-smoker; 1 = non-smoker; hypertension, 0 = absent, 1 = present; diabetes, 0 = absent, 1 = present; CKD, 0 = absent, 1 = present. Statistically significant results are highlighted in bold.

## Data Availability

The original contributions presented in the study are included in the article, further inquiries can be directed to the corresponding author.
